# Examining the relationship of sociodemographic factors, neighborhood cohesion and abnormal sleep duration among U.S. foreign-born subpopulations in the National Health Interview Survey

**DOI:** 10.1186/s12889-022-13523-z

**Published:** 2022-06-01

**Authors:** Kevin Villalobos, Francisco A. Montiel Ishino, Timothy S. McNeel, Faustine Williams

**Affiliations:** 1 Division of Intramural Research, National Institute on Minority Health and Health Disparities, National Institutes of Health, Bethesda, 7201 Wisconsin Avenue, Suite 533, Rockville, MD 20814 United States of America; 2 Information Management Services Inc., Calverton, MD United States of America

**Keywords:** Neighborhood cohesion, Sleep duration, U.S. foreign-born, Acculturation

## Abstract

**Background:**

Limited studies have examined the relationship of neighborhood cohesion and sleep duration between U.S. foreign-born Hispanics/Latinos and non-Hispanics/Latinos.

**Methods:**

We conducted a multinomial logistic regression using the 2013-2018 National Health Interview Survey on U.S. foreign-born adults ≥18 (*N* = 27,253). The outcome variable, sleep duration, was categorized as short sleep (≤6 hours), normal sleep (7 to 8 hours), and long sleep (≥9 hours). Neighborhood cohesion was categorized using tertiles (low, medium, high) from self-reported Likert scores. Our model included sociodemographic factors (i.e., age, marital status), socioeconomic status (i.e., education, employment status), health risk behaviors (i.e., body mass index, smoking status, alcohol drinking status), ethnic identity (i.e., Mexican, Puerto Rican, Cuban, Dominican, Central or South American, other/multiple Hispanic/Latino, and non-Hispanic/Latino), and acculturation factors (i.e., years lived in the U.S.; the language of interview).

**Results:**

Participants reporting low and medium neighborhood cohesion compared to high neighborhood cohesion had 45% (95% confidence interval [CI]:1.33-1.58) and 15% (95%CI:1.05-1.26) increased odds of short sleep (≤6 hours), compared to normal average sleep. Mexican participants had decreased odds of experiencing short sleep (adjusted odds ratio [AOR] = 0.82, 95%CI:0.73-0.92), while Puerto Ricans had increased odds of experiencing short sleep (AOR = 1.25, 95%CI:1.03-1.51) compared to non-Hispanics/Latinos.

**Conclusion:**

Neighborhood cohesion was associated with increased odds of short sleep duration. Social determinants, acculturation, and behavioral risk factors in the context of neighborhood cohesion are critical to understand U.S. foreign-born Hispanic/Latino sleep duration, as these factors may negatively synergize to exacerbate risk, worsening mental and physical health outcomes.

## Background

Abnormal sleep, that is, short or long sleep duration in the United States (U.S.) is an emerging epidemic that has been associated with poor physiological and mental health outcomes. Negative outcomes associated with abnormal sleep duration include cardiovascular disease, diabetes mellitus, inflammation, and obesity [1–3]. Abnormal sleep has also been associated with increased mortality [4]. And while about 35% of the U.S. population suffers from short sleep [5], minority groups are reported to have a higher prevalence of short sleep when compared to non-Hispanic Whites [6, 7] that may further widen the health disparities gap.

The U.S. Hispanic/Latino population will reach approximately 111 million by 2060 [8], making them the fastest growing minority group in the country. Health disparities among this group are prevalent in the U.S., with studies having reported that Hispanic’s/Latino’s general and mental health are associated with overall negative outcomes when compared to that of non-Hispanic Whites [9]. Several complex and interconnected determinants of health at individual, interpersonal, community and societal levels, coupled with the process of immigration and acculturation contribute to negative health outcomes that may synergize to worsen physical and mental health among U.S. foreign-born [10, 11]. A systematic review that explored the relationship between sleep and racial/ethnic disparities in cardiovascular disease found that minorities, in comparison to non-Hispanic Whites, were more likely to experience sleep deficiency that potentially increases the probability of developing or dying from this disease [12]. A possible explanation was that racial/ethnic minorities were more likely to reside in low-income/underserved neighborhoods or communities with poor cohesion where environmental stressors and hazards are common. These exposures ultimately increase their risk of cardiovascular disease and other adverse health outcomes [12]. Other factors including occupational stressors, access to treatment, and psychosocial stressors (e.g., perceived discrimination), are also known to contribute to the increased risk of cardiovascular disease among minorities [12].

Using the Behavioral Risk Factor Surveillance System (BRFSS) data, Grandner et al. [13], examined the social and behavioral factors of short sleep duration and found that socioeconomic and sociodemographic factors (e.g., income, education, employment, and sex) were associated with insufficient sleep [13]. Similarly, longer-than-average sleep or long sleep has also been associated with socioeconomic status (SES), obesity, and mental health disorder [14]. Using a nationally representative sample, Grandner et al. [15], further analyzed sleep complaints in approximately 160,000 adults in the U.S. and found that lower SES was associated with an increased prevalence of sleep complaints [15]. Moreover, ecological factors like neighborhood cohesion have also been associated with sleep [16].

Neighborhood cohesion is defined as the connectivity and social relationship among members of a community based on norms, values, and cultures that impact a neighborhood [17]. This relationship is not just based on ethnic homogeneity or SES, rather, a spectrum of ecological factors. High neighborhood cohesion has been associated with superior health outcomes. For instance, one study that explored neighborhood cohesion and sleep outcomes found that those reporting low neighborhood cohesion were more likely to have shorter sleep duration [16].

Hispanics/Latinos represent the largest immigrant group in the U.S. Subsequently, their health constitutes a significant proportion of the U.S. public health burden, as they represent more than 18% of the population [18]. Existing evidence shows that sleep duration among U.S. Hispanics/Latinos varies by socioeconomic, sociodemographic, place of residence, and acculturation (i.e., adaption to the host culture) factors [19]. Despite the importance of neighborhood environment, acculturation, and their effect on sleep duration, few studies have investigated the relationship between abnormal sleep and neighborhood cohesion among diverse, foreign-born Hispanic/Latino subpopulations in the U.S.

The purpose of this study was to examine sleep duration and neighborhood cohesion among U.S. foreign-born subpopulations, specifically Hispanics/Latinos compared to non-Hispanics/Latinos. We hypothesized that individuals living in a low cohesion neighborhood would experience abnormal sleep durations, that is short and long sleep. We also comprehensively examined the relationship of abnormal sleep and SES, health risk behaviors, and acculturation. While previous studies have examined the associations between neighborhood cohesion and short sleep duration among Hispanics/Latinos, our study not only included short sleep but long sleep duration as well. We analyzed complex survey data from the 2013-2018 National Health Interview Survey (NHIS) to fill a major gap in the current literature on sleep duration, neighborhood cohesion, Hispanic/Latino ethnicities, and health disparities.

## Methods

### Study participants

Data from the 2013-2018 NHIS were analyzed (see https://www.cdc.gov/nchs/nhis/index.htm). The NHIS is a cross-sectional household interview survey that targets the civilian noninstitutionalized population of the U.S. These analyses were restricted to foreign-born adults, i.e., those who were born outside of the 50 U.S. states and Washington, DC. Respondents with missing values for any of the variables of interest were excluded (i.e., 4,789), resulting in a final sample size of 27,253.

### Outcome variable

#### Sleep duration

Sleep duration was categorized using the following question: “On average, how many hours of sleep do you get in a 24-hour period? Enter hours of sleep in whole numbers, rounding 30 minutes (0.5 hour) or more up to the next whole hour and dropping 29 or fewer minutes.” Responses were categorized as ≤6 hours for short sleep, 7 to 8 hours for normal average sleep, and ≥9 hours for long sleep [20], where normal average sleep was used as the reference group.

### Predictor variable

#### Neighborhood cohesion

Neighborhood cohesion was based on NHIS participants’ agreement with the following four statements: “(1) this is a close-knit neighborhood, (2) there are people you can count on in this neighborhood, (3) people in this neighborhood can be trusted, and (4) people in this neighborhood help each other out. Values were assigned to the responses as follows: definitely agree = 1, somewhat agree = 2, somewhat disagree = 3, definitely disagree = 4.” The sum of the four values was subtracted from 17 to create a neighborhood cohesion score ranging from 1 to 13. Tertiles were created based on the weighted distribution of the neighborhood cohesion score among foreign-born adults, and categorized as low, medium, and high. Low and medium cohesion were compared to high neighborhood cohesion.

### Statistical analysis

Multinomial logistic regression models were used to examine the association between neighborhood cohesion and sleep duration. Sleep duration was the response variable, where normal average sleep (7 to 8 hours) was the reference category. Model 1 adjusted for the sociodemographic characteristics: sex (male [reference group], female), age (i.e. 18-44, 45-54, 55-64, 65-74, ≥75 [reference group]), marital status (single/never married [reference group], married/living with partner, divorced/separated, widowed), education (less than high school [reference group], high school graduate/GED, some college/associate degree, bachelor's degree or more), and employment status (employed, not employed [reference group]). Model 2 included the following health risk factors in addition to the sociodemographic characteristics: body mass index (BMI) in kg/m^2^ (<18.50, 18.50-24.99[reference group], 25.00-29.99, ≥30.00), alcohol drinking status (lifetime abstainer [≤12 drinks in life] [reference group], former drinker [no drinks past year], current drinker [1+ drinks past year]), and smoking status (never [reference group], former, current). In Model 3, the following ethnic identity and acculturation factors were also added: national Hispanic/Latino origin or ancestry (Mexican, Puerto Rican, Cuban, Dominican, Central or South American, other/multiple Hispanic/Latino, and non-Hispanic/Latino [reference group]), years lived in the U.S. (<10 years [reference group], ≥10 years), and the language of interview (English, Spanish, English and Spanish, and other [reference group]). Descriptive analyses were run on SAS software, version 9.4. Multinomial logistic regression analyses were weighted using Taylor series linearization methods to account for the stratified, multistage, cluster sample design of the NHIS [21] on SUDAAN 11.0.3.

## Results

### Sample characteristics

As seen in Table 1, the largest subpopulation among the sample was foreign-born non-Hispanic/Latino (50.4%), followed by Mexican (27.0%), Central or South American (11.6%), Puerto Rican (4.3% ), Cuban (3.6%), Dominican (2.4%), and lastly other/multiple Hispanic/Latino (0.7%). The majority of the participants (63.2%) reported a daily average sleep duration of 7 to 8 hours, while 30.5% reported that they slept less than or equal to 6 hours and 6.4% slept more than or equal to 9 hours. Regarding neighborhood cohesion, the largest proportion was found in the medium tertile of cohesion (37.7%), followed by high neighborhood cohesion (34.1%), and lastly low neighborhood cohesion (28.2%). About half of the participants reported being current drinkers (54.7%) and 75.0% reported that they have never smoked. When examining the number of years lived in the U.S., 81.0% reported that they have lived in the U.S. for 10 years or more. Regarding the language of the interview, the majority (71.0%) had the interview in English. See Table 1 for more details.

**Table 1:** Characteristics of U.S. Foreign-Born Adults Aged ≥18 Years by Hispanic Ethnicity, National Health Interview Survey, 2013-2018, *N* = 27,253

	**Mexican** (27.0%)		**Puerto Rican** (4.3%)		**Cuban** (3.6%)		**Dominican** (2.4%)		**Central or South American** (11.6%)		**Other/Multiple Hispanic/Latino** **(**0.7%)		**Non-Hispanic/Latino** (50.4%)		**All** (100%)	
**Characteristics**	**N**	**%**	**N**	**%**	**N**	**%**	**N**	**%**	**N**	**%**	**N**	**%**	**N**	**%**	**N**	**%**
**Sleep Duration Per Day**																
≤6 hours	1,968	27.0	443	37.4	271	27.3	242	33.6	997	31.2	65	32.3	4,422	31.6	8,408	30.5
7 to 8 hours	4,854	65.7	623	54.3	636	64.6	373	59.2	2,009	63.5	114	61.2	8,440	62.6	17,049	63.2
≥9 hours	532	7.2	102	8.3	76	8.1	35	7.1	160	5.3	16	6.5	875	5.9	1,796	6.4
**Neighborhood Cohesion**																
Low	2,519	33.6	446	36.5	280	30.2	264	41.1	1,157	35.6	64	32.6	3,246	22.5	7,976	28.2
Medium	2,714	37.0	387	34.8	300	33.0	230	35.5	1,175	37.4	61	32.9	5,301	38.8	10,168	37.7
High	2,121	29.4	335	28.7	403	36.9	156	23.4	834	27.0	70	34.5	5,190	38.7	9,109	34.1
**Sex**																
Male	3,371	50.8	473	50.0	468	53.7	244	45.0	1,379	49.8	81	47.9	6,335	48.1	12,351	49.2
Female	3,983	49.2	695	50.0	515	46.3	406	55.0	1,787	50.2	114	52.1	7,402	51.9	14,902	50.8
**Age Category**																
18 – 44	4,175	56.6	430	40.9	273	35.0	264	44.0	1,706	56.3	75	42.6	6,282	47.0	13,205	50.0
45 – 54	1,515	22.8	177	16.5	181	20.4	139	23.5	620	20.4	35	17.8	2,440	19.8	5,107	20.6
55 – 64	849	12.2	203	15.7	156	17.1	107	17.9	409	13.2	36	19.4	2,062	16.0	3,822	14.8
65 – 74	548	5.7	229	17.1	172	12.8	85	8.4	279	6.5	29	12.2	1,724	10.4	3,066	9.0
≥75	267	2.7	129	9.9	201	14.6	55	6.3	152	3.6	20	8.0	1,229	6.7	2,053	5.6
**Marital Status**																
Single/Never Married	1,164	15.3	269	19.7	142	15.3	139	21.1	647	18.1	34	11.8	2,695	16.3	5,090	16.4
Marred/Living with Partner	4,868	72.8	502	56.0	519	63.4	286	55.6	1,848	67.2	105	67.8	8,230	70.5	16,358	69.6
Divorced/Separated	975	8.7	273	16.6	196	13.9	169	17.9	525	11.3	35	14.5	1,742	8.5	3,915	9.6
Widowed	347	3.2	124	7.7	126	7.4	56	5.5	146	3.4	21	5.9	1,070	4.7	1,890	4.3
**Education**																
Less than High School	4,235	55.0	395	28.8	251	21.8	256	34.3	1,077	30.5	48	18.3	1,384	9.3	7,646	25.6
High School Graduate/GED	1,677	23.9	307	29.1	314	33.0	158	25.4	733	24.9	48	27.4	2,476	18.3	5,713	21.6
Some College/Associate’s	958	14.1	294	25.3	214	21.2	137	24.7	711	23.3	45	24.4	3,171	22.3	5,530	20.4
Bachelor’s Degree/More	484	7.0	172	16.9	204	24.0	99	15.6	645	21.2	54	29.9	6,706	50.1	8,364	32.4
**Employment Status**																
Employed	4,642	65.3	480	47.5	482	56.5	365	63.8	2,123	68.7	105	61.8	8,357	63.5	16,554	63.8
Not Employed	2,712	34.7	688	52.5	501	43.5	285	36.2	1,043	31.3	90	38.2	5,380	36.5	10,699	36.2
**BMI Category**																
<18.50, Underweight	63	0.7	12	0.9	10	1.1	9	2.3	32	1.2	4	1.7	433	2.6	563	1.8
18.50 – 24.99, Normal	1,767	23.3	349	30.3	322	30.1	225	31.0	1,035	31.4	60	29.3	6,546	46.5	10,304	37.0
25.00 – 29.99, Overweight	3,109	43.3	437	38.3	426	44.4	252	42.5	1,299	42.4	75	42.3	4,569	34.7	10,167	38.5
≥30.00, Obese	2,415	32.7	370	30.5	225	24.4	164	24.2	800	25.0	56	26.6	2,189	16.2	6,219	22.6
**Alcohol Drinking Status**^**a**^																
Lifetime Abstainer	2,487	31.7	410	34.0	449	40.3	250	36.3	1,076	32.9	64	28.3	4,729	35.1	9,465	34.1
Former Drinker	1,032	13.5	200	17.5	109	10.7	75	10.0	438	13.7	28	12.0	1,352	9.2	3,234	11.2
Current Drinker	3,835	54.7	558	48.5	425	49.0	325	53.8	1,652	53.5	103	59.6	7,656	55.7	14,554	54.7
**Smoking Status**																
Never	5,659	76.8	760	65.4	743	74.7	531	80.0	2,443	77.1	142	75.9	10,002	74.1	20,280	75.0
Former	979	13.9	226	20.2	135	15.4	77	12.9	504	16.3	37	14.6	2,349	16.5	4,307	15.8
Current	716	9.3	182	14.4	105	9.9	42	7.0	219	6.5	16	9.4	1,386	9.4	2,666	9.2
**Language of Interview**																
English Only	2,957	41.1	822	73.0	235	26.5	301	45.6	1,558	52.4	147	79.6	12,874	93.6	18,894	71.0
Spanish Only	2,860	38.2	202	17.4	662	60.9	270	42.1	1,125	32.0	28	10.3	24	0.3	5,171	17.4
Spanish and English	1,531	20.6	142	9.5	83	11.5	79	12.3	476	15.3	20	10.1	25	0.2	2,356	8.4
Other	6	0.1	2	0.1	3	1.1	0	0.0	7	0.3	0	0.0	814	6.0	832	3.2
**Years Lived in the U.S.**																
<10 Years	799	10.6	182	19.0	203	23.8	113	21.7	615	19.8	23	13.7	3,247	22.7	5,182	19.0
≥10 Years	6,555	89.4	986	81.0	780	76.2	537	78.3	2,551	80.2	172	86.3	10,490	77.3	22,071	81.0

All percentages are weighted

^a^Lifetime abstainer (<12 drinks in life); Former drinker (no drinks past year); Current drinker (1+ drinks past year)

### Multinomial logistic regression

#### Short sleep duration

As indicated in Model 1, we found that factors such as neighborhood cohesion, sex, marital status, education, and employment were associated with reporting short sleep. In Model 2, health risk behaviors including BMI and alcohol drinking status were associated with short sleep duration. Model 3, the most comprehensive model, was selected for interpretation.

### Short sleep duration comprehensive model

Compared to males, females had higher odds of reporting short sleep duration (adjusted odd ratio [AOR] = 1.13, 95% confidence interval [CI]: 1.04-1.22). See Table 2. Additionally, participants with low neighborhood cohesion and medium neighborhood cohesion, relative to those reporting high neighborhood cohesion, had higher odds of experiencing short sleep duration when compared to normal sleep (AOR = 1.45, 95% CI: 1.33-1.58 and AOR = 1.15, 95% CI: 1.05-1.26, respectively). Marital status was significantly associated with short sleep duration. Divorced/separated individuals (AOR = 1.39, 95% CI: 1.21-1.59) were more likely than single/never married to report short sleep duration. No statistically significant association was found between married/living with partner or widowed and short sleep duration, compared to those who were never married. A positive association was seen with being employed (AOR = 1.14, 95% CI: 1.04-1.25) and short sleep duration in comparison to normal sleep duration as well as having some college/associate degree (AOR = 1.18, 95% CI: 1.04-1.34) relative to less than high school individuals. Compared to aged ≥75 years, almost all age groups were not significant. However, aged 18-44 years (AOR = 0.83, 95% CI: 0.69-0.99) was statistically associated with short sleep duration when compared to normal sleep duration.

**Table 2:** Odds ratios and confidence intervals for associations between covariates and short sleep duration among U.S. foreign-born adults aged ≥18 years, from models 1-3, with normal sleep duration as reference, National Health Interview Survey, 2013-2018, *N* = 27,253

	**Short Sleep Duration (**≤6 **Hours) Adjusted Odd Ratios**								
	**Model 1**			**Model 2**			**Model 3**		
	**95% CI**			**95% CI**			**95% CI**		
	**AOR**	**Lower**	**Upper**	**AOR**	**Lower**	**Upper**	**AOR**	**Lower**	**Upper**
Intercept	0.38	0.31	0.47	0.32	0.26	0.40	0.40	0.29	0.54
**Neighborhood Cohesion**									
Low	**1.43**	**1.31**	**1.56**	**1.41**	**1.29**	**1.54**	**1.45**	**1.33**	**1.58**
Medium	**1.14**	**1.04**	**1.25**	**1.14**	**1.04**	**1.25**	**1.15**	**1.05**	**1.26**
High	ref.	-	-	ref.	-	-	ref.	-	-
**Sex**									
Male	ref.	-	-	ref.	-	-	ref.	-	-
Female	**1.09**	**1.01**	**1.18**	**1.13**	**1.04**	**1.22**	**1.13**	**1.04**	**1.22**
**Age category**									
18 – 44	**0.73**	**0.62**	**0.87**	**0.74**	**0.62**	**0.89**	**0.83**	**0.69**	**0.99**
45 – 54	0.87	0.73	1.04	0.86	0.72	1.04	0.93	0.77	1.12
55 – 64	1.07	0.89	1.28	1.04	0.87	1.26	1.09	0.91	1.32
65 – 74	0.97	0.82	1.15	0.96	0.81	1.14	0.98	0.83	1.16
≥75	ref.	-	-	ref.	-	-	ref.	-	-
**Marital Status**									
Single/Never Married	ref.	-	-	-	-	-	-	-	-
Married/Living with Partner	1.02	0.92	1.13	1.00	0.90	1.11	1.00	0.90	1.11
Divorced/Separated	**1.45**	**1.26**	**1.66**	**1.39**	**1.21**	**1.60**	**1.39**	**1.21**	**1.59**
Widowed	1.15	0.94	1.40	1.13	0.92	1.37	1.11	0.91	1.35
**Education**									
Less than High School	ref.	-	-	ref.	-	-	ref.	-	-
High School Graduate/GED	0.99	0.89	1.10	1.01	0.91	1.13	0.91	0.81	1.03
Some College/Associate Degree	**1.32**	**1.18**	**1.47**	**1.35**	**1.21**	**1.51**	**1.18**	**1.04**	**1.34**
Bachelor’s Degree or More	1.04	0.95	1.15	**1.11**	**1.01**	**1.23**	0.95	0.84	1.08
**Employment Status**									
Employed	**1.14**	**1.04**	**1.25**	**1.15**	**1.05**	**1.26**	**1.14**	**1.04**	**1.25**
Not Employed	ref.	-	-	ref.	-	-	ref.	-	-
**BMI Category**									
<18.50, Underweight				1.06	0.83	1.37	1.05	0.82	1.35
18.50 – 24.99, Normal				ref.	-	-	ref.	-	-
25.00 – 29.99, Overweight				**1.11**	**1.03**	**1.21**	**1.14**	**1.05**	**1.24**
≥30.00, Obese				**1.31**	**1.19**	**1.43**	**1.36**	**1.24**	**1.49**
**Smoking Status**									
Never				ref.	-	-	ref.	-	-
Former				1.08	0.98	1.20	1.07	0.96	1.19
Current				1.15	1.02	1.30	1.12	0.99	1.26
**Alcohol Drinking Status**^**a**^									
Lifetime Abstainer				ref.	-	-	ref.	-	-
Former Drinker				**1.14**	**1.01**	**1.28**	**1.15**	**1.03**	**1.30**
Current Drinker				0.99	0.91	1.08	1.00	0.92	1.09
**Hispanic Ethnicity**									
Mexican							**0.82**	**0.73**	**0.92**
Puerto Rican							**1.25**	**1.03**	**1.51**
Cuban							0.88	0.71	1.10
Dominican							1.07	0.87	1.32
Central or South American							0.97	0.87	1.11
Other/Multiple Hispanic/Latino							0.97	0.64	1.47
Non-Hispanic/Latino							ref.	-	-
**Years Lived in the U.S.**									
<10 Years							ref.	-	-
≥10 Years							**1.16**	**1.05**	**1.29**
**Language of Interview**									
English Only							**0.77**	**0.62**	**0.96**
Spanish Only							**0.64**	**0.50**	**0.82**
English and Spanish							**0.68**	**0.52**	**0.88**
Other							ref.	-	-

^a^Lifetime abstainer (≤12 drinks in life); Former drinker (no drinks past year); Current drinker (1+ drinks past year)

Health risk factors such as having an overweight BMI (AOR = 1.14, 95% CI: 1.05-1.24) and obese BMI (AOR = 1.36, 95% CI: 1.24-1.49) had increased odds of reporting short sleep when compared to normal BMI participants. The participants that reported being former drinkers (AOR = 1.15, 95% CI: 1.03-1.30) had increased odds of short sleep.

Ethnic identities such as Hispanic/Latino ethnicity were found to have an association with sleep duration. Those that self-identified as Mexican had decreased odds of experiencing short sleep (AOR = 0.82, 95% CI: 0.73-0.92) in comparison to foreign-born non-Hispanics/Latinos, while Puerto Ricans had increased odds of experiencing short sleep (AOR = 1.25, 95% CI: 1.03-1.51) in comparison to foreign-born non-Hispanics/Latinos. Acculturation factors such as those that lived in the U.S. for 10 years or more had increased odds of having experienced short sleep (AOR = 1.16, 95% CI: 1.05-1.29) in comparison to those that have lived in the U.S. for less than 10 years. For the language of interview, those that had the interview in English only (AOR = 0.77, 95% CI: 0.62-0.96), Spanish only (AOR = 0.64, 95% CI: 0.50-0.82), as well as both English and Spanish (AOR = 0.68, 95% CI: 0.52-0.88) had decreased odds of short sleep in comparison to other language. See Table 2 for more details.

#### Long sleep duration

Table 3 shows the relationship between neighborhood cohesion and long sleep duration. All the sociodemographic factors are shown to be associated with long sleep duration (see Model 1). We observed a statistically significant association between participants age, marital status, having a bachelor’s degree or higher, and being employed with long sleep duration. However, no statistically significant association was found between neighborhood cohesion and long sleep duration among U.S. foreign-born subpopulations aged ≥18 years using the NHIS data from 2013 to 2018. In Model 2 after adding health behaviors, we found that individuals who reported having underweight BMI (<18.50), obese BMI (≥30.00), and former drinkers had an increased odds of experiencing long sleep duration. Again, Model 3 was selected for interpretation.

**Table 3:** Odds ratios and confidence intervals for associations between covariates and long sleep duration among U.S. foreign-born adults aged ≥18 years, from models 1-3, with normal sleep duration as reference, National Health Interview Survey, 2013-2018, *N* = 27,253

	**Long Sleep Duration (≥9 Hours) Adjusted Odd Ratios**								
	**Model 1**			**Model 2**			**Model 3**		
	**95% CI**			**95% CI**			**95% CI**		
	**AOR**	**Lower**	**Upper**	**AOR**	**Lower**	**Upper**	**AOR**	**Lower**	**Upper**
Intercept	0.61	0.46	0.80	0.48	0.36	0.65	0.50	0.32	0.77
**Neighborhood Cohesion**									
Low	0.94	0.80	1.11	0.93	0.79	1.10	0.94	0.79	1.11
Medium	0.86	0.73	1.00	0.86	0.74	1.01	0.86	0.74	1.01
High	ref.	-	-	ref.	-	-	ref.	-	-
**Sex**									
Male	ref.	-	-	ref.	-	-	ref.	-	-
Female	**1.18**	**1.03**	**1.37**	**1.23**	**1.06**	**1.43**	**1.24**	**1.07**	**1.44**
**Age category**									
18 – 44	**0.34**	**0.28**	**0.43**	**0.35**	**0.28**	**0.43**	**0.33**	**0.26**	**0.41**
45 – 54	**0.28**	**0.22**	**0.37**	**0.28**	**0.22**	**0.37**	**0.28**	**0.21**	**0.36**
55 – 64	**0.29**	**0.22**	**0.38**	**0.28**	**0.22**	**0.37**	**0.28**	**0.21**	**0.37**
65 – 74	**0.43**	**0.34**	**0.55**	**0.43**	**0.33**	**0.55**	**0.42**	**0.33**	**0.54**
≥75	ref.	-	-	ref.	-	-	ref.	-	-
**Marital Status**									
Single/Never Married	ref.	-	-	ref.	-	-	ref.	-	-
Married/Living with Partner	**0.69**	**0.58**	**0.82**	**0.68**	**0.57**	**0.81**	**0.68**	**0.58**	**0.82**
Divorced/Separated	**0.69**	**0.54**	**0.89**	**0.66**	**0.52**	**0.85**	**0.67**	**0.52**	**0.86**
Widowed	**0.61**	**0.46**	**0.81**	**0.60**	**0.45**	**0.80**	**0.61**	**0.46**	**0.80**
**Education**									
Less than High School	ref.	-	-	ref.	-	-	ref.	-	-
High School Graduate/GED	0.96	0.80	1.15	0.97	0.81	1.17	0.97	0.80	1.17
Some College/Associate Degree	0.84	0.69	1.03	0.86	0.70	1.04	0.86	0.69	1.07
Bachelor's Degree or More	**0.59**	**0.49**	**0.71**	**0.62**	**0.51**	**0.75**	**0.62**	**0.49**	**0.77**
**Employment Status**									
Employed	**0.54**	**0.46**	**0.64**	**0.54**	**0.46**	**0.64**	**0.55**	**0.47**	**0.65**
Not Employed	ref.	-	-	ref.	-	-	ref.	-	-
**BMI Category**									
<18.50, Underweight				**1.91**	**1.27**	**2.88**	**1.88**	**1.26**	**2.79**
18.50 – 24.99, Normal				ref.	-	-	ref.	-	-
25.00 – 29.99, Overweight				1.02	0.87	1.20	1.02	0.87	1.20
≥30.00, Obese				**1.38**	**1.16**	**1.64**	**1.38**	**1.16**	**1.64**
**Smoking Status**									
Never				ref.	-	-	ref.	-	-
Former				1.10	0.92	1.32	1.11	0.92	1.33
Current				1.16	0.92	1.46	1.16	0.92	1.46
**Alcohol Drinking Status**^**a**^									
Lifetime Abstainer				ref.	-	-	ref.	-	-
Former Drinker				**1.25**	**1.01**	**1.53**	**1.25**	**1.01**	**1.54**
Current Drinker				1.08	0.92	1.27	1.08	0.91	1.27
**Hispanic Ethnicity**									
Mexican							1.09	0.89	1.33
Puerto Rican							1.16	0.85	1.58
Cuban							1.07	0.73	1.57
Dominican							1.12	0.55	2.27
Central or South American							0.87	0.67	1.14
Other/Multiple Hispanic/Latino							0.99	0.51	1.93
Non-Hispanic/Latino							ref.	-	-
**Years Lived in the U.S.**									
<10 Years							ref.	-	-
≥10 Years							0.84	0.70	1.02
**Language of Interview**									
English Only							1.13	0.81	1.60
Spanish Only							1.00	0.68	1.47
English and Spanish							1.28	0.83	1.97
Other							ref.	-	-

^a^Lifetime abstainer (≤12 drinks in life); Former drinker (no drinks past year); Current drinker (1+ drinks past year)

### Long sleep duration comprehensive model

In our comprehensive analysis (i.e., Model 3), we assessed acculturation factors while controlling for sociodemographic and health behavior factors (see Table 3). Our results showed that females had increased odds of reporting long sleep duration (AOR = 1.24, 95% CI: 1.07-1.44) when compared to males. Similarly, individuals who reported being married/living with partner, divorced/separated, and widowed were less likely to experience long sleep (AOR = 0.68, 95% CI: 0.58-0.82, AOR = 0.67, 95% CI: 0.52-0.86, AOR = 0.61, 95% CI: 0.46-0.80, respectively) compared to never married participants. Additionally, we found that those who reported having bachelor’s degree or more had decreased odds of experiencing long sleep duration relative to those with less than high school education (AOR = 0.62, 95% CI: 0.49-0.77). Also, participants who were employed had decreased odds of experiencing long sleep (AOR = 0.55, 95% CI: 0.47-0.65) in comparison to those unemployed. Further, a statistically significant association was also found between all age categories and long sleep duration compared to age 75 and older.

Regarding health behavior risk factors, having an obese or underweight BMI was associated with higher odds of experiencing long sleep duration in comparison to normal weight (AOR = 1.38, 95% CI: 1.16 -1.64, AOR = 1.88, 95% CI: 1.26 -2.79; respectively). When examining alcohol drinking status, former drinkers had increased odds of experiencing long sleep duration (AOR= 1.25, 95% CI: 1.01-1.54) in comparison to lifetime abstainers. No statistically significant relationship exists between neighborhood cohesion, Hispanic/Latino ethnicity, acculturation factors (i.e., years lived in the U.S. and the language of interview) and long sleep duration. See Table 3 for more details.

## Discussion

To our knowledge, this study is amongst the first to examine the relationship between neighborhood cohesion and sleep duration among U.S. foreign-born (i.e., Hispanics/Latinos and non-Hispanics/Latinos) that accounted for SES, health risk behaviors, and acculturation. We found that low and medium neighborhood cohesion were strongly associated with short sleep duration among U.S. foreign-born adults. Furthermore, our study also found that the odds of abnormal sleep duration varied among foreign-born Hispanic/Latino subpopulations. Specifically, we found that Mexicans were 18% less likely to report short sleep, while Puerto Ricans had 25% increased odds of short sleep duration. These Hispanic/Latino subpopulation differences could be explained not only by neighborhood cohesion, but by SES, behavioral risk factors, and acculturation factors in context of neighborhood environments, which have been reported to affected sleep duration among U.S. foreign-born adults.

Using NHIS data on 13,537 participants from 2010-2015, Murillo et al. [22], assessed the relationship between normal versus short sleep duration and neighborhood cohesion by Hispanic/Latino subgroups. The authors reported significant associations between medium and high neighborhood cohesion and normal sleep for all Hispanic/Latino subgroups, but the strongest association was among Cubans/Cuban Americans. These associations were accounted for by assessing sociodemographic and socioeconomic factors (i.e., sex/gender, education, employment status, marital status) in the model. Dudley et al. [23], investigated sleep patterns in diverse U.S. Hispanic/Latino populations using the Hispanic Community Health Study/Study of Latinos (HCHS/SOL). The results indicated that individuals with Mexican heritage experienced the longest sleep duration while Puerto Ricans had the shortest sleep duration. Conversely to Murillo et al. [22], they only accounted for sleep outcomes (i.e., sleep duration hours, sleep fragmentation index, sleep maintenance efficiency) in their model. On the other hand, using the NHIS, Garcia et al. [24], reported that except for U.S.-born Cubans, all Hispanic/Latino subgroups had higher odds of short sleep duration compared to non-Hispanic Whites in the U.S. Our study did not find any relationship between Cuban, Dominican, Central or South American, and other/multiple Hispanic/Latino ethnicity and sleep duration among foreign-born adults in the U.S., when compared to foreign-born non-Hispanics/Latinos. Garcia et al. [24] accounted for demographic characteristics, acculturation measures, socioeconomic characteristics, and behavioral health characteristics (e.g., age, sex, marital status, the language of interview, education status, smoking status) in their model.

Sociodemographic factors in context of the neighborhood were found to have a significant relationship to neighborhood cohesion and sleep duration. We must also acknowledge that individual level factors also play a role in sleep duration and neighborhood dynamics. For instance, sex/gender has been reported to affect both short and long sleep duration [25–28]. Earlier research has suggested that poor neighborhood conditions were positively associated with higher relative risk of reporting very short sleep, long sleep, as well as napping in both men and women [28]. A systematic review that explored sleep characteristics across the lifespan in individuals from the Netherlands, United Kingdom, and the U.S., found that women were more likely to experience short sleep or slightly less efficient sleep than men [27]. A review that focused on sleep and women’s health associated menstruation, pregnancy (i.e., prenatal & postnatal), and menopause as contributors to abnormal sleep among women due to the hormonal changes that occur within these physiological events [29]. In contrast, a study that analyzed the American Time Use Survey, a nationally representative sample from 2003 to 2007 (*N* = 56,149), and examined gender differences in time for sleep, [26] revealed women were more likely to sleep better than men, although the difference was small. Our study revealed that U.S. foreign-born women were 13% and 24% more likely than men to experience both short and long sleep duration, respectively. While abnormal sleep in female participants was more prevalent in our study, research assessing sleep patterns in males and females was limited, specifically long sleep. Further research on factors contributing to abnormal sleep as well as its variation among males and females is necessary.

Regarding socioeconomic factors, our study found that employed individuals had 14% higher odds of short sleep and 45% lower odds of long sleep. Similarly, individuals with some college or associate’s degree were more likely to report short sleep (18%) while those with bachelor’s degree or more were less likely to experience long sleep duration (38%) in comparison to those that had less than a high school education. We observed a non-statistically significant association between attaining a bachelor’s degree or higher and short sleep duration in U.S. foreign-born adults. Although it has been reported that individuals with higher educational attainment have an association with lower rates of chronic diseases compared to those with less education levels, higher educational attainment does not translate to improved sleep outcomes in our sample. However, prior studies on sleep duration found that having higher education was associated with better sleep outcomes [15, 28]. Using the Jackson Heart Study on 5,301 African American participants, Johnson et al. [30], investigated the association between individual-level socioeconomic position, which was measured by income and education, and neighborhood characteristics with sleep duration and sleep quality. The authors reported an association between lower individual socioeconomic position and sleep duration/quality, that is, individuals with lower socioeconomic position were associated with increased odds of long sleep and poorer sleep quality. The authors also found that higher neighborhood problems were associated with poor sleep duration/quality [30]. More research, especially prospective studies, is necessary to provide a better understanding of the impact of socioenvironmental factors on sleep.

Acculturation factors such as years lived in the U.S. are essential when analyzing sleep duration among U.S. foreign-born subpopulations. The present study indicated that those that have lived in the U.S. for 10 years or more have 16% higher odds of short sleep duration. A potential cause is the adoption of U.S. health risk behaviors that have conflicting effects. An example would be an increase of high caloric foods that lack nutritional value resulting in an increase in BMI. We found that those that were overweight or obese had higher odds of short sleep. Additionally, those that were obese and underweight had increased odds of long sleep duration. Individuals that have adopted or are adapting to the American diet are more likely to experience abnormal sleep duration [31–33]. A study that explored short sleep duration, BMI, and appetite regulatory hormones (e.g., leptin and ghrelin) found that individuals that were considered short sleepers had an elevated ghrelin level and reduced leptin level, which was associated with increased BMI [34, 35]. Another study that examined the association between sleep duration and dietary consumption of fruits/vegetables reported that among adults in the United Kingdom, those that consumed lower amounts of fruits/vegetables were more likely to experience abnormal sleep [36].

While the language of interview was associated with short sleep, that is, English and/or Spanish compared to other language, this may be indicative of the diverse linguistic spectrum among U.S. foreign-born Hispanics/Latinos. Furthermore, this may be the result of a diverse mix from both highly acculturated and non-acculturated foreign-born adults in the U.S. While we cannot make global assumptions about acculturation using language of interview, we did use it as a proxy. The assumptions regarding acculturation were limited in our study, as such future studies must go beyond language used for interview to capture language usage in specific everyday situations.

Of note was that long sleep duration was not found to have a significant relationship with neighborhood cohesion for U.S. foreign-born adults. Prior studies have found that long sleep duration was associated with cardiovascular disease, obesity, and diabetes, [2] but our study did not find association between neighborhood cohesion and long sleep duration. To our knowledge, while this study was not the first to explore neighborhood cohesion and abnormal sleep duration, it is amongst the first to explore the concept of long sleep among the U.S. foreign-born and its association with neighborhood cohesion.

Our study, however, was not without limitations. First, the NHIS data are cross-sectional, which does not allow us to assess changes in short and long sleep duration over time or establish and understand the causal link between neighborhood cohesion and abnormal sleep duration. Second, the number of acculturation variables in the dataset was limited. Third, other language was used as the reference for “language of survey” due to non-Hispanic/Latino being the largest group. Consequently, the purpose of our study was not only to examine Hispanic/Latino subgroups in the U.S. but also foreign-born non-Hispanic immigrant groups. Fourth, the sleep duration measures were self-reported which could have been biased. Despite these challenges, our findings are consistent with previous studies and contribute to the body of knowledge regarding the effect of neighborhood cohesion and abnormal sleep among immigrants and Hispanic/Latino subpopulations. Overall, our results demonstrate that there was an association with low and medium neighborhood cohesion and short sleep duration among U.S. foreign-born.

## Conclusion

The dynamic changes and growth in U.S. immigrant populations call for innovative efforts to ensure their health and well-being. Immigrant sleep duration has only recently been explored and needs to be a priority in the public health system in the U.S. Sleep duration has many components that will affect its outcome, such as health risk behaviors, SES, acculturation factors, ethnic identity, and neighborhood cohesion. This study found that sleep duration varies among U.S. foreign-born Hispanics/Latinos when compared to foreign-born non-Hispanics/Latinos. Future directions will extensively analyze U.S. foreign-born subpopulations independently to understand risk factors of abnormal sleep duration, as well as explore why specific subgroups are experiencing poorer outcomes, e.g., Puerto Rican’s short sleep duration. Ultimately, it is essential to examine and promote interventions that will improve neighborhood cohesion and overall sleep quality, as well as health. Our study provides another facet to the burgeoning literature to help create salient sleep interventions for U.S. foreign-born individuals and promote health equity.

## Data Availability

The datasets generated and/or analyzed during the current study are available in the Nation Health Interview Survey repository, https://www.cdc.gov/nchs/nhis/data-questionnaires-documentation.htm

## Abbreviations

BMI: Body Mass Index
BRFSS: Behavior Risk Factor Surveillance System
HCHS: Hispanic Community Health Study
NHIS: National Health Interview Survey
SES: Socioeconomic Status
SOL: Study of Latinos
US: United States
